# A case management of hypertension in the elderly in sub-Sahara Africa: lessons from Granny

**DOI:** 10.11604/pamj.2017.26.165.10660

**Published:** 2017-03-21

**Authors:** Ahmadou Musa Jingi, Liliane Mfeukeu Kuate, Jean Jacques Noubiap

**Affiliations:** 1 Department of Medicine and Specialties, Faculty of Medicine and Biomedical Sciences, University of Yaoundé 1, Yaoundé, Cameroon; 2 Cardiology Unit, Department of Internal Medicine, Yaoundé Central Hospital, Yaoundé, Cameroon; 3 Department of Medicine, Groote Schuur Hospital and University of Cape town, Cape Town, South Africa; 4 Medical Diagnostic Centre, Yaoundé, Cameroon

**Keywords:** Hypertension, management, elderly, side effects, sub-Saharan Africa

## Abstract

Management of chronic disease conditions in the elderly is challenging. They usually have many co-morbidities requiring multiple drug regimens, and memory or cognitive problems that can interfere with management. Also, they sometimes have a degree of social problems as they might often live alone, and thereby cater for their daily activities with minimal assistance. Multiple drug use combined with their fragile health predispose them to adverse drug reactions, drug-drug interactions, and direct drug toxicity from overdosing. We report and discuss the lessons learnt from the case of an elderly woman in an urban setting in sub-Saharan Africa who presented with problems of drug dosing, adverse drug effect, and drug-drug interaction that might prove useful in the future management of hypertension with angiotensin converting enzyme inhibitors.

## Introduction

Management of chronic disease conditions in the elderly is challenging. They usually have many co-morbidities requiring multiple drug regimens, and memory or cognitive problems that can interfere with management [1]. Also, they sometimes have some degree of social problems as they might often live alone, and thereby cater for their daily activities including the intake of medicines with minimal assistance [2]. Multiple drug use combined with their fragile health predisposes them to adverse drug reactions, drug-drug interactions, and direct drug toxicity from potential overdosing [3]. They may even sustain physical injuries with eventual poor outcomes as a result of medicine use [4–6]. We report and discuss the lessons learnt from the case of an elderly woman in an urban setting in sub-Saharan Africa, who presented with problems of drug dosing, adverse drug effect, and drug-drug interaction that might prove useful in the future management of hypertension with angiotensin converting enzyme inhibitors (ACEIs).

## Patient and observation

Madame H, is a 74-year-old woman with long-standing hypertension managed with the angiotensin converting enzyme inhibitor perindopril 5mg daily for over 13 years. She also had osteoarthritis of the shoulders, wrists, and knees for which she was not taking any specific medicines. She had limited social assistance in the management of her conditions as no one was making sure she was taking her medication correctly. However, she was compliant to her anti-hypertensive treatment with good blood pressure control, until she developed Quincke’s edema which was attributed to perindopril as it was her only medicine at that time. Her treatment was immediately switched to the calcium channel blocker amlodipine 5mg daily, and a short course of an anti-histamine cetirizine 10 mg daily to manage the conspicuous labial-facial edema. With subsequent visits, she was found to be taking perindopril in place of amlodipine (due to stock out) alongside cetirizine from the previous prescription. Amazingly, no labial-facial edema occurred. Both medicines were stopped and she was put on amlodipine 5mg daily, with poor control of her blood pressure on subsequent visits. Her treatment was switched to a fixed drug combination of amlodipine 5mg plus indapamide 1.5mg (thiazide-like diuretic) daily, with optimal blood pressure control for her age. Subsequently, she developed the conspicuous labial-facial edema. Investigation revealed she had a stock out of her fixed drug anti-hypertensive and reverted to perindopril 5mg (old stock not discarded). She was switched back to the fixed anti-hypertensive medicine. She also complained of neck, shoulder and wrist pain with electrical discharges for which high dose vitamin B (2 tablets twice daily) was prescribed. With subsequent visit, she complained of posterior neck and scalp pain, and clinical evaluation was remarkable for an unusually low sitting blood pressure reading of 104/67 mmHg on the right arm (control arm), for a regular pulse of 74 beats per minute. She was not in acute distress. Investigation revealed she was taking four times the prescribed anti-hypertensive medicine (2 tablets twice daily totaling 20mg of amlodipine and 3mg of indapamide daily), alongside the high dose vitamin B (2 tablets twice daily).

**Ethics approval and consent to participate:** The report of this case was approved by the Institutional Review Board of the Yaoundé Central Hospital, Cameroon. The patient provided written informed consent. The patient consented for the publication of her case in the form of a scientific paper.

## Discussion

This case highlights some of the challenges in managing chronic disease conditions in the elderly. Many lessons (old and new) have been learnt from this case and warrants sharing and further investigations. Firstly, the anti-histamine (cetirizine) appeared to reduce the risk of Quincke’s edema. Secondly, extreme caution should be exercised when adding or switching medicines in the elderly. Thirdly, the number and the frequency of intake of a pill can influence the number and the frequency of intake of another pill, which might be potentially toxic. Fourthly, late adverse drug reaction to the ACEIs can occur up to thirteen years of medicine use. Frequent side effects associated with the use of ACEIs family of anti-hypertensive medicines are intractable non-productive cough and the conspicuous labial-facial angio-edema or Quincke’s edema [3, 7]. When any of these occur, the drug and related medicines are immediately stopped and black listed for the patient. Switching to another medicine with comparable efficacy such as the angiotensin receptor blockers is an option, but often carries a higher cost and low availability for treating a chronic condition in low-income settings [8]. This case suggests that anti-histamines can reduce the risk of frequent adverse events such as angio-edema associated with the use of ACEIs, despite the fact that it is bradykinin mediated [9]. However, this observation has to be studied further in a randomized double blind placebo-controlled trial.

Drug overdose, whether intentional or accidental in the context of poly-pharmacy is also frequent in daily clinical practice [1]. As anecdotes, a 52-year old man treated for heart failure was concomitantly taking two ACEIs at full dose as the result of a switch from a more expensive to a less expensive ACEI, until he developed intractable non-productive cough shortly after he started both medicines. Also, a 74 year old man had to take over thirty medicines including steroid injections daily, cumulated from various consultations from many physicians until he developed iatrogenic Cushing syndrome and multiple organ damage. When switching to another medicine in the elderly, we should ensure that the old and/or frequently used medicines are discarded to reduce the risk of drug intoxication or drug-drug interaction [3]. Also, we should limit our prescription to the most needed medicines, and choose from the once daily intake or the most simple drug regimens [10]. In this case, adding twice daily vitamin B (that can be forgone) erroneously induced this elderly woman to quadruple her anti-hypertensive medicine with the risk of hypotension and falls [5, 6, 11]. Patients on ACEIs, especially the elderly, should monitored regularly for late onset adverse drug reactions [7].

## Conclusion

This case highlights some of the challenges in managing chronic diseases in the elderly. This case suggests that, the anti-histamine (cetirizine) can reduce the risk of Quincke’s edema. Also, extreme caution should be exercised when adding or switching medicines in the elderly. The number and the frequency of intake of a pill can influence the number and the frequency of intake of other pills taken at the same time, which might be potentially toxic in the elderly. Adverse drug reactions to ACEIs can occur years after the onset of use.
